# Sustaining energetic communities: energy citizenship and participation in an age of upheaval and transition

**DOI:** 10.1038/s41598-024-53367-8

**Published:** 2024-02-08

**Authors:** Breffní Lennon, Niall Dunphy

**Affiliations:** 1 Cleaner Production Promotion Unit, School of Engineering and Architecture, University College Cork, Cork, Ireland; 2 Environmental Research Institute, University College Cork, Cork, Ireland

**Keywords:** Environmental social sciences, Sustainability

## Abstract

The human use of energy is inherently understood and experienced through socially constructed frameworks. However, the degree of engagement with this topic on the part of humanities and the social sciences has until recently been uneven at best. This seems strange given current upheavals experienced in Europe and across the globe as the climate and biodiversity crises deepen. At the centre of all these crises is the energy system. Energy flows through various forms of natural and social circuitry (from production, to distribution and consumption) and these *energyscapes* are sited at the local, national, and transnational scales. The correlation between the (meta)physical flows taken by the various forms of energy we depend on—and the transitory social, cultural, economic, and political relationships that frame them—require much deeper study if we are to achieve the types of sustainable communities envisaged by the United Nations as part of its sustainable development goals (SDGs) for 2030. Arising from a review of current literature, this article presents recent research into the forming of citizen energy communities in Europe and the governance structures designed to facilitate their development. It also highlights the key drivers and barriers to citizen engagement with emergent, novel energetic communities.

## Introduction

The evolution of electricity systems has, over the course of the last century, seen a shift from small-scale, often highly-localised networks to the rolling out of regional and national grids that prioritised large-scale generation. This was further underscored by centrally controlled transmission and distribution infrastructure, typically owned and operated by state-run organisations and/or large business operators^1^ functioning as oligopolies if not outright monopolies. This remains the case in parts of North America most notably Texas, Alaska and Québec. These systems allowed for the provision of relatively affordable electricity to a majority of the population, while also aiding industrial development and adding to socio-economic wellbeing. However, this arrangement required (nor facilitated) little or no citizen participation beyond that of ‘the consumer’, who in turn is framed by the market parameters of demand and supply, with ‘engagement’ measured in terms of affordability^2^. This conception of participation has begun to change with citizens now expected to take on more active roles, particularly as prosumers^3^ or what has been euphemistically termed the ‘active consumer’^4,5^.

Also, while social sustainability (SS)—and by extension sustainable communities (SCs)—has been part of development discourses since the 1980s, globally there are still rather mixed interpretations as to their meaning. Indeed, in the case of SCs they are still rarely defined^6^. However, this has not stopped it framing recent developments in urban renewal^7,8^, (eco)tourism^9–11^, higher education^12^, food systems^13^ and in response to the COVID-19 pandemic^14^. Interestingly, Visa et al.^15^ align it to the nearly Zero Energy Community concept most often applied to energy consumption in buildings^15^. Indeed, the one area where we have seen progress has been around energy with the advent of the European Commission’s Clean Energy for All Package (CEP), which positions the role of citizens and communities more firmly within a sustainable energy system. The legislative framework, as laid out in two key directives (the revised Internal Electricity Market Directive (EU) 2019/944^16^ which introduces the term ‘citizen energy communities’; and the revised Renewable Energy Directive (EU) 2018/2001^17^, which sets out the framework for ‘renewable energy communities’), categorise community-oriented energy initiatives under the common umbrella term—‘energy community’—and outlines the two variants it prioritises: notably ‘citizen energy communities’ (CECs) and ‘renewable energy communities’ (RECs)^18^. In addition, the introduction of the European Green Deal in 2020 has seen a systematic review of existing laws in relation to climate, while also introducing new legislation on biodiversity, agriculture, building renovation, and the circular economy, with the overarching aim of achieving climate neutrality within the European Union by 2050^19^.

Although similar efforts are underway elsewhere, the most coherent expression of these goals remains with the EU. Indeed, the development of renewable technologies and distributed energy systems is already having a significant impact on the electricity industry landscape^20^. Beyond the infrastructural changes the evolution of renewable energy systems provide an opportunity to apply local governance frameworks to energy production, particularly in terms of energy self-sufficiency at both the community and regional levels^21^. As such, energy flows through various forms of natural and social circuitry (from production, to distribution and consumption) and these *energyscapes* are sited at the local, national, and transnational scales. Consequently, a deeper understanding of the correlation between the (meta)physical flows taken by the various forms of energy we depend on and the transitory social, cultural, economic, and political relationships that frame them is needed if we are to achieve the types of sustainable communities envisaged by the United Nation’s sustainable development goals (SDGs) for 2030. This article is prepared in the context of the ACCEPT project that is exploring the sustainable energy communities concept in its development of demand response digital tools^22^, and the EnergyMeasures project that is working to address energy poverty across 7 European countries^23^ and highlights recent research into the forming of citizen energy communities (CECs) in Europe and the governance structures designed to facilitate their development. It also highlights the drivers and barriers to citizen engagement with emergent, novel energetic communities.

## Results

With energy communities set to play a key role in moving to a low-carbon economy, citizens across Europe are seeing their expected roles transition from merely passive consumers to more active, prosumer-oriented activities comprising hosting/operating local generation infrastructure, participating in associated demand response programmes, and/or contributing to improving the energy efficiency of the built environments they negotiate daily. Drawing from the review of the literature conducted for the ACCEPT Horizon 2020 project, this section outlines a typology of drivers and barriers to citizen participation in CECs across Europe. How consumers are encouraged to participate in the energy transition and the governance and socio-political factors that condition these engagements is already impacting the European Union’s energy transition effort.

Indeed, CECs can offer members a vehicle to plan, finance, and own their own energy systems, as well as to take a more proactive role in managing their energy services. In working collectively, citizens can restructure existing power networks and achieve better levels of distribution, diversity, and social inclusivity, particularly in terms of energy. Though this potential remains under-utilised by national governments to date. Building on our own research and work by Robinson and Stephen^24^, Table 1 summarises the key governance and socio-political factors that condition the levels of citizen engagement in existing CECs. Indeed, it should be noted that many of these factors often act as a barrier to greater CEC formation across the European Union.

**Table 1:** Governance and socio-political factors that condition citizen engagement in CECs^25^.

Uncertainty and change in renewable energy policies	Regulatory gaps, inconsistencies, complicated tax rules, and changing regulations such as the closure of Feed-in Tariff (FIT) schemes have a significant impact on market confidence both for investors and for community groups tasked with setting up a CEC
Lack of institutional support	Non-recognition of CECs as legal identities, changing conditions from regulators, and complex administrative procedures related to planning permissions
Limited funding and access to finance	There is some early-stage funding to assess CECs technical feasibility in some jurisdictions. However, projects are often stalled due to limited, or poorly targeted funding opportunities resulting in perceptions of poor financial viability for CEC projects
Lack of support for innovation	Lack of access to technical expertise, information, and data to design, plan, implement and commission a project also impacts project viability
Lack of organisational capacity and time	CECs often rely on volunteers who have limited access to good quality information and/or time to develop greater individual and organisational capacity building
Exclusion of vulnerable populations from projects	High initial investment and time costs make CEC membership rather exclusive and more typically attract middle- or high-income individuals, and therefore (un)intentionally ostracise certain cohorts of society
Lack of trust in the private and public sectors	Lack of trust in local and national governments, and existing energy developers, has led some community organisations to miss out on opportunities to leverage industry partnerships, which are often critical for accessing funding and knowledge sharing

However, the many barriers to greater citizen participation in community energy projects can be addressed through improved government and sectoral supports. This remains a significant challenge, however, especially for those operating in countries with rapidly changing policy landscapes or with diminishing state subsidy schemes. Consequently, the financial and legislative supports designed to promote CECs must continue to adapt to changing political and market conditions. In addition, all emerging policy approaches and innovations should prioritise the ‘community-led’ aspects to low carbon development if we are to encourage greater levels of citizen participation in the energy transition^24^. The main drivers for citizen engagement and participant in CECs are outlined in Table 2 below.

**Table 2:** Drivers of citizen engagement in CECs^25^.

Improved subsidy support	Increased promotion of investments in renewables by passing laws governing community and local ownership in the energy sector, introducing a mixture of grants and tax advantages to the community energy landscape
Increased institutional support	Higher institutional recognition of time and capacity requirements of CEC members, and simplified planning processes
Early-stage funding and access to alternative forms of finance	Funding delivered in a way that ensures early-stage funding to enable the development of innovative technologies and new business models and for project consolidation
Better information and knowledge sharing	Greater attention to delivering capacity building such as advice, one-stop-shops, learning about new business models, and technical knowledge to allow communities to make informed decisions and access new opportunities
Increasing local cohesion and sense of community	Promote policies that strengthen local cohesiveness and interpersonal trust within communities to increase citizens’ willingness to contribute to the community and participate in CECs
Fostering new partnerships	Supporting partnerships among citizens, government, local authorities, industry organisations, and commercial developers to overcome technical and funding barriers
Support new technical innovations and alternative business models	Look into flexibility services and emerging new approaches to energy production, transfer, and consumption. Innovations include battery storage, demand side management (DSM), demand side response (DSR), flexibility services, peer-to-peer (P2P) energy trading, vehicle to grid (V2G), smart grids and energy systems

As we can see from Tables 1 and 2, energy is very much a socio-technical system with deep social and technical inertias that continue to hamper the pace of the energy transition. For instance, the legislative and regulatory contexts at national and regional level often remain oriented towards maintaining existing centralised models, with incumbent utility companies dominating energy production and distribution. However, more decentralised models involving geographically dispersed, small-scale generation units located closer to consumers do offer an alternative^26^. For instance, greater decentralisation supported by a strengthening of regional-level transmission infrastructure can help deal with issues like peak load demand and renewable energy intermittency and aggregation within different geographical scales^27^. Indeed, decentralised energy production that is underscored by community-oriented organisation and deployment is seen as a leading pathway to foster the Energy Transition among Europe’s seemingly disparate populations^28,29^.

## Discussion

While the concept of ‘community energy’ has its basis in discourses on sustainability, good self-governance, and energy independence^30^, it is the institutional contexts within which members of energy communities interact that have the most significant impact on the types of energyscapes that will ultimately be formed. Therefore, a more sustainable—and sustaining—energy system involves a social transformation that is both deep and systemic, and supported by the work of citizens^31^. Suitably supported community energy projects offer a perfect opportunity to combine individual and public interests into a more mutually beneficial socio-technical system than has heretofore been the case^32^.

Current energy transition trajectories still have numerous barriers that tend to offset the potential drivers to citizen participation—or indeed, consumer engagement—when forming CECs in most European countries^33,34^. Though new legislative frameworks being established in member states will help to counter these barriers, experience demonstrates that unintended or unforeseen consequences often arise when legislation is interpretated at different scales. Often resulting in significant deviations from the initial vision of the authors of the policy in question. Adapting a multi-level perspective, and a clear willingness to proactively engage with stakeholders is required. As Howlett and Ramesh^35^ suggest “decisions by governments to retain the status quo are just as much policy as are decisions to alter it”. This is an important point, given all policy extends beyond the deliberate choices made by official actors in the policy domain—the so-called “realm of potential choices” (*ibid*.)—and are subject to highly complex, changing (re)alignments of governments and the societal actors affected.

Despite the clear benefit to tackling the climate crisis, the grid integration of variable renewable energy (VRE) sources does present major challenges to the energy system. Resolving this issue requires deploying a range of simultaneous and integrated solutions, most notably demand response^36^. Energy communities are a promising organisational vehicle for involving citizens in addressing issues around residential demand. These can be both through formal and/or informal citizen-led structures that collectively facilitate the local deployment of energy technologies. Evidence indicates that deploying RES technologies, while also being sensitive to locally specific social contexts, can greatly enhance the social acceptance of such technologies at the local level^37^ and indeed encourage public participation in decision-making on vital infrastructure^38,39^.

Though, as recent EU legislation has framed it, energy communities do not necessarily have to involve energy technologies per se. According to Caramizaru and Uihlein^40^ an ‘energy community’ can refer to any number of citizens who participate in the energy system on a collective basis. As such, energy communities are nothing new^28^. Prior to the establishment of national grids, energy communities were often characterised in terms of their geographic isolation and distance to each other with individual cities and towns operating their own electricity networks, either through privately owned companies or municipal utilities. The Great Recession of 2008^41^ appears to have acted as somewhat of a catalyst for the emergence of energy communities consisting of individuals and groups seeking more affordable, environmentally friendly, and locally controlled energy systems^42^. Energy cooperatives remain the most common way in which citizens participate and/or take ownership of renewable energy. However, other forms are also evident including limited partnerships, development trusts, and foundations. Indeed, Caramizaru and Uihlein identify at least 24 different approaches taken by energy communities representing a diverse range of governance structures, activities, and legal forms^40^.

Energy communities are often represented, particularly in popular media, as possessing a democratising effect on power, offering members of such organisations unique opportunities to plan, finance, (co-)own and/or manage their own energy services^43–45^. They can potentially challenge existing top-down energy structures and transform passive consumers of energy into active co-producers, giving power and control back to the people^42^. As energy citizens, working collectively in an energy community, they can positively impact the energy sector, helping to restructure electric power networks in favour of greater levels of distribution, diversity, and inclusivity^2,46,47^. Community energy projects can be categorised several ways. From a technological perspective, initiatives may address the supply side of energy (e.g., via solar and wind power projects); or the demand side, applying energy conservation measures, awareness-raising initiatives, etc.^48^. Initiatives may also be distinguished by their socio-cultural characteristics. These can range from projects that are very much citizen-oriented, with clear decision-making capacity assigned to members (e.g., energy cooperatives), to initiatives that involve citizens (e.g., government- or business-led projects) but have little decision-making power assigned to those citizens involved.

The full impact of community energy on the energy system has not been fully realised to date. However, the potential for energy communities to become a standard model in energy markets across the bloc can certainly be seen from recent developments taking place in a number of member states^28^. In the Netherlands, for example, over 500 local energy initiatives are active today, while in Germany over half of all newly installed RES production capacity involves ownership on the part of citizens as opposed to commercial interests alone. Usually, these take the form of individual citizen investors, farmers, energy cooperatives, etc. In fact, Kampman et al.^49^ estimate some 83% of EU households (approximately 187 million homes) could potentially become an energy citizen, contributing to RES production, demand response and/or energy storage. It is also becoming increasingly common to see energy communities take on new roles as providers of energy and/or offering specific, highly specialised energy services. In addition to generation, these can range from energy sharing and distribution of energy, to electro-mobility and other energy services^40^.

How energy communities are coalescing at different governance scales also provides scholars with new opportunities to develop further insights into the energy transition. Melville et al*.*’s^50^ paper, for example, examines the potential of applying a commons-based framework to urban energy management using as a case study community-oriented approaches to electricity demand response in a UK city. While acknowledging the considerable literature already available on smart grids, particularly in terms of systemic value^51,52^, demand response criteria^53–56^, issues around privacy^57,58^ and community-based approaches^59–61^, conceptualising energy (notably electricity) as a form of commons remains under-theorised and open for further examination.

The ‘community’ aspect to many community energy groups very often relates to certain locales^62^ or highly localised understandings of place^63^. Community, for some, is both contentious and contested both in terms of how it is represented and interpreted by different stakeholders^64^. Consequently, it has been used for numerous rhetorical and ideological ends either to legitimise or delegitimise an opponent’s policy position and recently as a way to offload governmental responsibilities^65^. The ascribing of community (see also citizen) terminology to somehow indicate legitimacy or a common ownership of a project has also been applied largescale energy projects across Europe. Drawing on Walker’s characterisation of the various meanings applied to the term^65^, we suggest that CECs can similarly be divided into six potential expressions that also reflect the key conceptual criteria set out in the Renewable Energy Directive (EU) 2018/2001. CECs, are best understood as operating along several axes, including.

As an *actor* with agency that offers citizens a vehicle whereby, they can generate, consume, and store energy, while also participating in energy markets and adding flexibility to the energy system. As an actor, concerned about climate change for example, a CEC can enable citizens to find solutions to through the production and consumption of RES, while also raising local awareness of the need to transition to low-carbon energy systems. At the same time, these solutions provide other social benefits at the local level, e.g., cheaper more reliable electricity infrastructure for remote populations and energy services that allow citizens to live more comfortably.

As *scale*, CECs can be seen as located somewhere between the individual and the local government, comprising of collective formations of citizens, societal organisations, and private actors that do not necessarily partake in formal government structures, but rather operate somewhat outside at a more autonomous level.

As *place*, whereby citizens define or interpret a CEC as operating along highly localised terms (e.g., through self-consumption using infrastructure that is not linked directly to the national grid).

As a *network*, formed by new relationships between different stakeholders beyond the local. These networks form partnerships between individuals, companies, societal organisations, and governments, with the aim of achieving joint benefits such as optimising the energy system.

As a *process*, CECs can be communities created with a bottom-up approach that is voluntary and where citizens participate in collaborative processes. Trust is an important element for maintaining cohesion during this process.

Finally, as an *identity*, CECs adopt ways of thinking and as such can be seen a means to improve the image of an area, e.g., being known as a place where ‘green energy’ is being produced. These identities emphasise collective interests or a ‘sense of community’ that go beyond the household and family, and even potentially beyond the normative boundaries of the state (e.g., online communities etc.).

### Motivations for citizen engagement

Similarly, in addition to their spacial contexts energy communities also occupy several temporal scales, most notably in terms of duration, with memberships continuously evolving over time. Institutional innovations, which energy communities can be considered, are taken up my different cohorts in society at different rates^66^. This can make it more difficult to identify any single causal factor in the success or failure of individual energy communities or the degree of engagement by citizens. Therefore, understanding the underlying motivations that influence energy community investors at the local and individual scales becomes more important. Recent research has begun to examine the factors influencing local participation in community energy projects^67–69^ which should assist decision-makers in designing more effective supporting policies.

The key drivers commonly accepted to motivate participation in community energy projects, include social, economic, environmental, and political factors^43,44^. Research has also suggested the importance of community identity, trust, and the normative social behaviours that condition expectations for establishing acceptance and/or a willingness to participate in community energy projects^70–75^. As Bauwens^66^ suggests this heterogeneity should be carefully considered by policymakers. A key motivating factor expressed to us for joining a CEC has been to make a more tangible contribution to addressing the climate crisis. Indeed, both the literature and our own research indicates that participation is often framed as a type of ‘journey’ which changes and evolves as the individuals’ own experiences and levels of knowledge change and evolve. There is also a clear correlation drawn between holding concern on environmental topics with the practicing of pro-environmental behaviour more generally^76^ and a greater willingness on the part of citizens to participate in community energy projects^68,69^.

Soeiro and Ferreira Dias^77^ set out what they see as being the most and least important motivations for people when considering joining an energy community^77^. They found that citizens tend to prioritise a range of motivating factors, from the most important:

- Concerns about environmental and climate impacts of traditional energy technologies.
- Participating in the energy transition.
- Influence in the community and trust.
- Local interaction within the community, bring people together.
- Be independent of the energy producers.
- Increase local acceptance for renewable energies.

To the least important:

- Local income generation.
- Change role of consumers in society.
- Reliable local energy supply.
- Lower energy costs.
- Local investment.

As this happens, citizens may turn to community energy projects if they see them as offering a strong social critique of traditional governmental and commercial energy interests, with members expressing greater trust in their own initiative and its ability to contribute to a more sustainable and equitable energy system^30^. Similarly, research undertaken by Bauwens^78^ when examining members of the Belgian cooperative found that individual motivations to invest and participate in the energy community could be explained through the innovation adoption perspective^79^. In their study, early members of the energy community were observed to attach more value to innovative renewable energy production than later members. Accordingly, interpersonal trust has also been found to support citizen participation and is considered in the literature to be an important factor in encouraging social cohesiveness and cooperation^42,80^. What can only be described as a certain reluctance on the part of policy makers to acknowledge the powerful role such motivating factors have on communities has resulted in policy cycles across Europe continue to struggle with encouraging wider participation and investment in community energy initiatives across diverse societal groups. Incorporating these findings into future policy cycles will be a significant first step on the path to implementing what should be a just and fair sustainable energy transition. As Schiavo^81^ suggests, all future strategic planning and policy frameworks must emphasise the central role of communities and success should only be measured using a common understanding ‘community engagement’ that moves away from tokenistic gestures and silencing of citizens’ voices that up until now has very often been the case.

## Conclusion

Communities more likely to be sustainable if they organise in terms of sustainable energy use. To facilitate this, more robust regulations around insulation, energy use and the built environment are still required^6,80^. Essentially, successful decarbonisation of the energy system will be driven by a combination of factors and synergies coalescing around technological development, policy exertion, and changing societal attitudes^19^. Unlike in previous energy transitions, this one cannot rely solely on technological development with the expectation that society can be coerced into following or adapting to new socio-technical orders. Rather, technology and its deployment will need to go hand-in-hand with robust policy enforcement (particularly over the short-term) that adheres to the justice concerns of citizens if we are to overcome the many barriers to realising the transformative change that is required. This paper highlights the importance of recognising and responding to existing drivers and barriers to citizen participation in energy, taking into account the real motivating factors that frame.

## Methods

The emerging field of ‘sustainability transitions’ offers a wealth of research material, including a plethora of conceptual frameworks and terminologies across a variety of academic disciplines^82^. The work presented in this article comprises mainly desk-based literature reviews of existing research from academic research databases and what is often termed ‘grey literature’—such as politico-legal documents, research reports, etc. Given the growing importance of citizen energy communities in the European Union this article emphasises the importance of the literature review for characterising the many drivers, limitations, and challenges faced by those engaged in forming community energy projects and the CECs that underpin them. A review of the literature is fundamental to the research process^83,84^ and by assessing existing knowledge and theoretical developments arising from practice-based analysis one can develop new knowledge, theories, and insights on a chosen topic^85^, comprising ‘the familiar iterative process of searching, reading, annotating, organising, summarising, analysing, and finally synthesising’^86^.

Therefore, the value of a thorough literature review to the research cannot be overestimated, whether for synthesising existing research or for integrating findings and analysis on a specific topic^87,88^. Without it, one cannot expect to develop a deeper understanding of the topic they are engaged with^89^. This obvious importance, however, has resulted in it being assigned more of a preliminary exercise or a precursor to ‘real’ research^90,91^. This is a mistake and largely misses to point as to why it is needed in the first place. Indeed, a literature review serves as a research method in its own right particularly for recognising both the gaps and potential insights when framing current research and planning for the future^92^.

The objectives of the literature review for our work in the project was twofold: (1) to map existing and emerging patterns of consumer engagement with demand response (DR) initiatives in the EU; and, (2) to identify the key factors driving citizen energy community formation, positioning them within their socio-economic, socio-cultural, and geographical contexts. Principal sources for academic literature included bibliographic databases available through university library subscriptions, or available for free online (e.g., open access journals). The material selected included academic material, European Union and national government legislation and reports, as well as ongoing reports from EU-funded research projects. While all major search engines will identify the majority of extant material available, each still have their strengths and weaknesses^93^. In order to overcome these weaknesses three search engines were selected: Science Direct (https://www.sciencedirect.com/), JSTOR (https://www.jstor.org/), and Google Scholar (https://scholar.google.com/).

The database searches comprised a Boolean keyword search that combined, or excluded, terms using the ‘Boolean operators’ AND, OR, NOT. This approach offered a certain adaptability to the searches, especially when used in combination. However, there is also a potential that the approach may yield either too many or too few results. When conducting searches involving the ‘social’ or ‘human’ dimension to energy, the often nebulous or contested assemblage of meaning ascribed to some terms (e.g., variability in the usage of some terminology in energy research more generally can be attributed to the diversity of disciplines studying the topic) presents its own problems. The emergent nature of much of the research reflects the emerging processes shaping the energy communities themselves^82^. Consequently, we chose to review a variety of articles to identify the most common keyword terms used in relation to energy communities. These were then applied in a systematic Boolean keyword search, in conjunction with a ‘backward’ and ‘forward’ snowballing process^83,94^ that augmented the Boolean keyword search. This provided extra depth to the research and ameliorated gaps that can arise due to variations in definitions of key terms and helped ensure relevant research was not inadvertently omitted.

Another notable part of the research informing this article involved assembling a representative sample of case studies across fourteen European countries, including the participating countries of the ACCEPT Horizon 2020 project representing a broad diversity of geographies, population compositions, economic development, and national renewable energy system landscapes^95^. Also, capturing a diverse range of national contexts was considered a priority to supplement findings applicability and to learn from the specific issues arising from the diverse contexts. The selection criteria defined a diversity of CEC formations comprising:

1. The scale of the energy community project.
2. The specific form take by the CEC.
3. Capturing the broad range of experiences across the fourteen diverse European countries.
4. The extent of available literature on individual projects.

### Analysis and interpretation

After gathering documents on the different case studies in the fourteen European countries, we engaged in a thematic analysis identifying several common themes from the literature, with corresponding examples from the case studies themselves^95^. Braun and Clarke^96^ consider thematic analysis a core method for qualitative analysis and allows practitioners to identify, analyse, organise, describe, and report on key themes from the data they engage with, while also complementing many other forms of qualitative analysis^97^. As a means for encoding qualitative information, it can also be considered a bridging tool between qualitative research and quantitative research^98^.

The expanded application of terminologies and their related linguistic variabilities—associated with concepts like *energy communities*—has received considerable attention in recent years particularly after the introduction of the European Clean Energy Package (CEP), along with the new definitions of renewable energy communities (RECs) and citizen energy communities (CECs). In addition, the transposition of the two directives described above into the legal frameworks of individual Member States has already added a diversity of interpretations at the national level^99^. To overcome variability, the range of relevant terms and descriptions commonly used as keywords included: ‘community energy’, ‘renewable energy communities’, ‘clean energy communities’, ‘citizen energy communities’, ‘energy cooperatives’, ‘sustainable communities’, ‘local energy communities’, and ‘community energy’. Secondary terms such as ‘demand response’, ‘energy storage’ were also used to assist in identifying relevant studies. From this we were able to outline the country-specific contexts for each country^95^, including their reaction to the EU legislation on climate change and energy, how the term ‘community energy’ is understood and applied, and a brief history of energy communities and/or citizen-led initiatives on energy and the associated challenges experienced in each country.

### Ethics approval and consent to participate

The research reported in this paper received ethical approval from the Social Research Ethics Committee (SREC) at University College Cork (Log no. 2022-146 and 2022-184). All research methods used have been carried out in accordance with the relevant guidelines and regulations, including the receipt of informed consent from all participants.

## Data Availability

Further information is available on the ACCEPT (https://www.accept-project.eu/) website, in the form of numerous project deliverables, newsletters and other public documents. Qualitative data from interviews, workshops etc. are not public in order to protect the anonymity of project participants.
